# Publisher Correction: Topological optical differentiator

**DOI:** 10.1038/s41467-021-22493-6

**Published:** 2021-04-07

**Authors:** Tengfeng Zhu, Cheng Guo, Junyi Huang, Haiwen Wang, Meir Orenstein, Zhichao Ruan, Shanhui Fan

**Affiliations:** 1 Department of Electrical Engineering, Ginzton Laboratory, Stanford University, Stanford, CA USA; 2 Interdisciplinary Center for Quantum Information, State Key Laboratory of Modern Optical Instrumentation, and Zhejiang Province Key Laboratory of Quantum Technology and Device, Department of Physics, Zhejiang University, Hangzhou, China; 3 Department of Electrical Engineering, Technion-Israel Institute of Technology, Haifa, Israel

**Keywords:** Applied optics, Optical materials and structures, Optical physics, Imaging techniques

Correction to: *Nature Communications* 10.1038/s41467-021-20972-4, published online 29 January 2021.

The original PDF version of this Article contained errors in Equations 3, which incorrectly read:

3 $${{\bf{e}}}_{{\bf{o}}{\bf{u}}{\bf{t}}}{\bf{R}}({k}_{x}=0,{k}_{y}=0){{\bf{e}}}_{{\bf{i}}{\bf{n}}}=0$$

The correct form of Eq. (3) is:

3 $${\bf{e}}^{\dagger }_{{\bf{o}}{\bf{u}}{\bf{t}}}{\bf{R}}({k}_{x}=0,{k}_{y}=0){{\bf{e}}}_{{\bf{i}}{\bf{n}}}=0$$

The PDF version also contained an error in the inline equation at the end of the first paragraph of the first Results’ subsection, which incorrectly read:

$$r({k}_{x},{k}_{y})={{\bf{e}}}_{{\bf{o}}{\bf{u}}{\bf{t}}}{\bf{R}}({k}_{x},{k}_{y}){{\bf{e}}}_{{\bf{i}}{\bf{n}}}$$

instead of

$$r({k}_{x},{k}_{y})={\bf{e}}^{\dagger }_{{\bf{o}}{\bf{u}}{\bf{t}}}{\bf{R}}({k}_{x},{k}_{y}){{\bf{e}}}_{{\bf{i}}{\bf{n}}}$$

Both in the PDF and html versions of the article, at the end of the first paragraph of the first Results’ subsection, in Equation 2 and in the inline equations after the sentence “The entire reflection process is then described by…”, the tilde notations should be exactly above the symbols “*S*”

Both in the PDF and html versions of the article, at the end of the first paragraph of the first Results’ subsection, the inline equation in the sentence starting with “which shows that the zero of…” should read:

$$r({k}_{x},{k}_{y})$$

rather than

$$rt({k}_{x},{k}_{y})$$

Both the pdf and the html have been updated to include these corrections.

