# Supplementary material for: Topological optical differentiator
Source: Nat Commun. 2021 Jan 29;12:680. doi: 10.1038/s41467-021-20972-4 (PMC7846860; doi:10.1038/s41467-021-20972-4)
Supplement: Supplementary file 1 — Supplementary Information [file 41467_2021_20972_MOESM1_ESM.pdf]

Supplementary Information for "Topological optical differentiator"

Tengfeng Zhu[1,2], Cheng Guo[1], Junyi Huang[2], Haiwen Wang[1], Meir Orenstein[3], Zhichao Ruan[2*], Shanhui Fan[1*]
[1] *Department of Electrical Engineering, Ginzton Laboratory, Stanford University, Stanford, CA 94305, USA*
[2] *Interdisciplinary Center for Quantum Information, State Key Laboratory of Modern Optical Instrumentation, and Zhejiang Province Key Laboratory of Quantum Technology and Device, Department of Physics, Zhejiang University, Hangzhou 310027, China*
[3] *Department of Electrical Engineering, Technion-Israel Institute of Technology, 32000 Haifa, Israel*

SUPPLEMENTARY NOTE 1: CALCULATION OF THE TRANSFER FUNCTION

Here, we calculate the form of transfer function $r\left(k_x, k_y\right)$ [Eq. (4) in the main text] and the corresponding C_x and C_y [Eqs. (9) and (10)]. In the derivation, we use similar notations and formulations following Ref. [34] of the main text, where one special case of cross polarization is discussed. We denote $\mathbf{e_{in}} = \left(e_{in}^x, e_{in}^y\right)^T$ and $\mathbf{e_{out}} = \left(e_{out}^x, e_{out}^y\right)^T$ as the normalized incident and output polarizations in the x-y plane, respectively.

For a spatial frequency component of the incident or output beam, we define its own s- and p-polarization basis vectors as $\hat{\mathbf{s}} = \hat{\mathbf{k}} \times \mathbf{n}$ and $\hat{\mathbf{p}} = \hat{\mathbf{s}} \times \hat{\mathbf{k}}$, where $\hat{\mathbf{k}} = \mathbf{k}/|\mathbf{k}|$ indicates the propagation direction of the spatial frequency component and $\mathbf{n}$ is the normal vector of the interface. Since spatial frequency components with different (k_x, k_y) have different propagation directions, their s- and p-polarization basis vectors vary with (k_x, k_y). In the s- and p-polarization bases for a spatial frequency component with (k_x, k_y), the incident (output) polarization can be written as $\mathbf{U_{1(2)}}\mathbf{e_{in(out)}}$, where

$$\mathbf{U_1} = \begin{pmatrix} 1 & \frac{\cot\theta_0}{k_0}k_y \\ -\frac{\cot\theta_0}{k_0}k_y & 1 \end{pmatrix}, \tag{1a}$$

$$\mathbf{U_2} = \begin{pmatrix} -1 & -\frac{\cot\theta_0}{k_0}k_y \\ -\frac{\cot\theta_0}{k_0}k_y & 1 \end{pmatrix}. \tag{1b}$$

Then the transfer function $r\left(k_x, k_y\right) = \mathbf{e_{out}^\dagger}\mathbf{R}\left(k_x, k_y\right)\mathbf{e_{in}}$ can be written as

$$r\left(k_x, k_y\right) = \mathbf{e_{out}^\dagger}\mathbf{U_2}^\dagger\tilde{\mathbf{R}}\left(k_x, k_y\right)\mathbf{U_1}\mathbf{e_{in}}. \tag{2}$$

Here, the matrix $\tilde{\mathbf{R}}\left(k_x, k_y\right)$ is

$$\tilde{\mathbf{R}}\left(k_x, k_y\right) = \begin{pmatrix} r_p & 0 \\ 0 & r_s \end{pmatrix}, \tag{3}$$

where r_p and r_s are the Fresnel reflection coefficients for p- and s-polarized plane waves at wavevector (k_x, k_y), respectively. In order to achieve the differential operation, $\mathbf{e_{in}}$ and $\mathbf{e_{out}}$ need to satisfy the cross-polarization condition [Eq. (3) in the main text], i.e. $r\left(k_x = 0, k_y = 0\right) = 0$. Based on Supplementary Equation (2), we have

$$-r_{p0}e_{in}^x {e_{out}^x}^* + r_{s0}e_{in}^y {e_{out}^y}^* = 0, \tag{4}$$

where r_{p0} and r_{s0} are the Fresnel reflection coefficients for p- and s-polarized plane waves at the incident angle θ_0, respectively.

By using Supplementary Equations (1-4) and expanding the Fresnel reflection coefficients to their first-order derivatives as $r_{p(s)} = r_{p0(s0)} + \frac{\partial r_{p(s)}}{\partial\theta}\frac{k_x}{k_0}$, we then have the transfer function calculated as

$$r\left(k_x, k_y\right) = \frac{1}{k_0}\left(-e_{in}^x {e_{out}^x}^* \frac{\partial r_p}{\partial\theta} + e_{in}^y {e_{out}^y}^* \frac{\partial r_s}{\partial\theta}\right)k_x - \frac{\cot\theta_0}{k_0}\left(e_{in}^y {e_{out}^x}^* + e_{in}^x {e_{out}^y}^*\right)\left(r_{p0} + r_{s0}\right)k_y. \tag{5}$$

* shanhui@stanford.edu; zhichao@zju.edu.cn

Therefore, the coefficients C_x and C_y in Eq. (4) have the following forms:

$$C_x = \frac{1}{k_0}\left(-e_{in}^{x} {e_{out}^{x}}^{*}\frac{\partial r_p}{\partial\theta} + e_{in}^{y} {e_{out}^{y}}^{*}\frac{\partial r_s}{\partial\theta}\right), \tag{6a}$$

$$C_y = -\frac{\cot\theta_0}{k_0}\left(e_{in}^{y} {e_{out}^{x}}^{*} + e_{in}^{x} {e_{out}^{y}}^{*}\right)\left(r_{p0} + r_{s0}\right). \tag{6b}$$

Specifically, in the case with a linear output polarization $\mathbf{e_{out}} = (-\sin\gamma, \cos\gamma)^T$, according to Supplementary Equation (4), the required input polarization can be written as

$$\mathbf{e_{in}} = N\begin{pmatrix} r_{s0}\cos\gamma \\ -r_{p0}\sin\gamma \end{pmatrix}, \tag{7}$$

where $N = 1\Big/\sqrt{\left|r_{s0}\cos\gamma\right|^2 + \left|r_{p0}\sin\gamma\right|^2}$ is a normalization factor. Then, Supplementary Equation (6) becomes

$$C_x = N\frac{\sin\gamma\cos\gamma}{k_0}\left(r_{s0}\frac{\partial r_p}{\partial\theta} - r_{p0}\frac{\partial r_s}{\partial\theta}\right), \tag{8a}$$

$$C_y = -N\frac{\cot\theta_0}{k_0}\left(r_{p0}\sin^2\gamma + r_{s0}\cos^2\gamma\right)\left(r_{p0} + r_{s0}\right), \tag{8b}$$

which is the Eq. (9) in the main text. In the case of operating at the Brewster angle with $r_{p0} = 0$ in Supplementary Equation (4), the output polarizer has an orientation angle $\gamma = \pi/2$ and hence selects the polarization $\mathbf{e_{out}} = (-1, 0)^T$. In this case, arbitrary $\mathbf{e_{in}} = (e_{in}^{x}, e_{in}^{y})^T$, rather than only the one in Supplementary Equation (7), can satisfy the cross-polarization condition [Eq. (3) in the main text]. Based on Supplementary Equation (6), the coefficients C_x and C_y in transfer function with $\mathbf{e_{in}} = (e_{in}^{x}, e_{in}^{y})^T$ and $\mathbf{e_{out}} = (-1, 0)^T$ become:

$$C_x = \frac{1}{k_0}\frac{\partial r_p}{\partial\theta}e_{in}^{x}, \tag{9a}$$

$$C_y = \frac{\cot\theta_0}{k_0}r_{s0}e_{in}^{y}, \tag{9b}$$

which is the Eq. (10) in the main text.

SUPPLEMENTARY NOTE 2: EXPERIMENTAL METHODS FOR MEASUREMENT OF THE MAGNITUDES OF TRANSFER FUNCTIONS AND THE TOPOLOGICAL CHARGES

We experimentally measure the magnitude of transfer function by measuring the output spatial spectrum of a beam and normalizing it with the incident one. The output spatial spectra when operating in the total internal reflection and at the Brewster angle are measured using the setups shown in Supplementary Figures 1(a) and 1(b), respectively, without the beam splitters in the dashed boxes. We use a solid-state laser at the wavelength of 532 nm with adjustable power (Changchun New Industries Optoelectronics Tech. CO. LTD, MGL-U-532) as the source. We use a lens (L1) to generate a focused incident beam in order to amplify the amplitudes of the plane wave components with higher spatial frequencies, which is advantageous for the measurement over a relatively broad spatial bandwidth. The focused beam at the back focal plane of L1 has a waist radius of 2.82 μm. We use a linear polarizer (P1) and a quarter-wave plate (QWP) to tune the polarization of the incident beam to be the required one, as discussed in the main text. When operating in the total internal reflection case [Supplementary Figure 1(a)], the incident beam propagates through the first lateral side of the prism at normal incidence and then reflected by the glass-air interface at an incident angle of 70.24°. When operating at the Brewster angle [Supplementary Figure 1(b)], the incident beam is directly reflected by the air-glass interface. After the light reflection, we use a second linear polarizer (P2) to select the required output polarization and use a lens L2 to perform optical Fourier transform on the reflected beam. Then, we measure the magnitude squared of the output spatial spectrum $[I_{out}(k_x, k_y)]$ with a CMOS camera (Thorlabs DCC1645C) at the

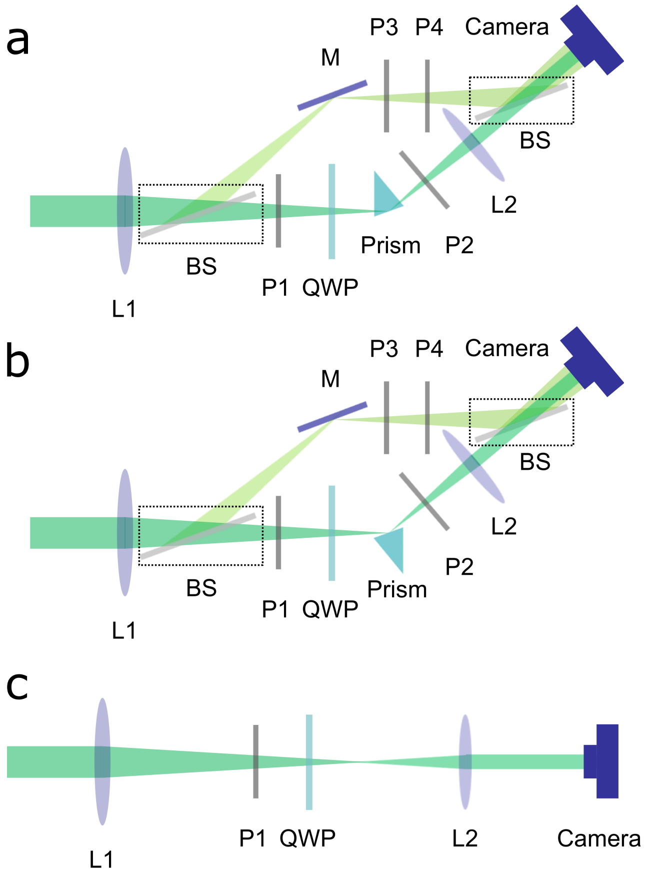

Supplementary Figure 1. Experimental setups for measuring the magnitudes of transfer functions and the topological charges. (a) Setups for measuring the output spatial spectrum (without the beam splitters in the dashed boxes) and the topological charges (with the beam splitters in the dashed boxes) when operating in total internal reflection at a glass-air interface. (b) Same as (a), except that we operate on the air-glass interface at the Brewster angle. (c) Setup for measuring the input spatial spectrum. The source is a laser at wavelength of 532 nm. The material of the prism is BK7. BS, beam splitter; P1, P2, P3 and P4, linear polarizers; QWP, quarter-wave plate; M, mirror; L1 and L2 are lenses with focal lengths of 60 mm and 50 mm, respectively.

back focal plane of L2, and also the intensity I_1 of the incident beam after the QWP. The corresponding magnitude squared of the incident spatial spectrum $[I_{in}(k_x, k_y)]$ is measured with the setup shown in Supplementary Figure 1(c), and the corresponding intensity I_2 of the incident beam after the QWP is also measured. The magnitude of transfer function can then be acquired by normalizing the output spatial spectrum with the corresponding incident one, as $r(k_x, k_y) = \sqrt{\frac{I_{out}(k_x,k_y)/I_1}{I_{in}(k_x,k_y)/I_2}}$.

To measure the topological charges, we use the first beam splitter (BS1) after L1 to generate a reference beam, and use the second beam splitter (BS2) to recombine the reference beam and the output beam for interference. Thus, the CMOS camera records the interference fringes between the output beam and the divergent reference beam. The polarizers P3 and P4 in Supplementary Figures 1(a) and 1(b) are used to achieve a high contrast in measured interference fringe patterns. Specifically, P4 is used for tuning the polarization of the reference beam to be the same as that of the output beam, and P3 is used as a tunable attenuator for the reference beam.

SUPPLEMENTARY NOTE 3: EXPERIMENTAL METHODS FOR ISOTROPIC EDGE DETECTION

In experimental demonstration of the isotropic edge detection, we use the setups in Supplementary Figures 2(a) and 2(b) to measure the input [Figs. 4(b) and 4(e) in the main text] and output images [Figs. 4(c) and 4(f) in the main text]. In both setups in Supplementary Figures 2(a) and 2(b), we send a collimated laser beam through the mask and an imaging system consisting of lenses L1 and L2, generating an image on a CMOS camera at the back focal plane of L2. The imaging system consisting of L1 and L2 has a demagnification factor of 10. Also, in both cases, the

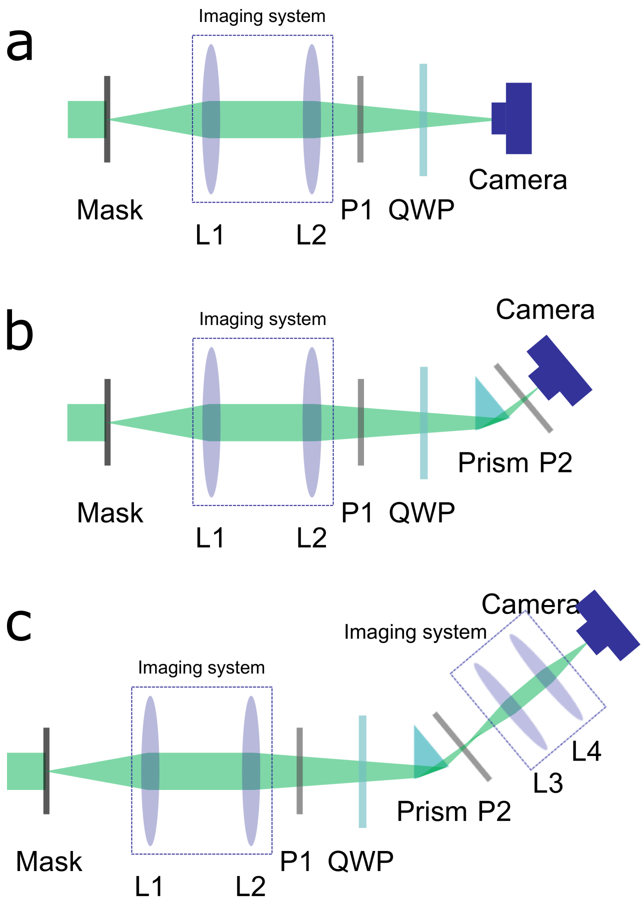

Supplementary Figure 2. Experimental setups for demonstration of edge detection when operating in the total internal reflection. (a) Setup for measuring the input images. (b) Setup for measuring the output images. (c) Setup with an additional imaging system consisting of L3 and L4 for magnification of the output images. The source is a laser at the wavelength of either 532 nm or 632.8 nm. The material of the prism is BK7. P1 and P2, linear polarizers; QWP, quarter-wave plate; L1, L2, L3 and L4 are lenses with focal lengths of 500 mm, 50 mm, 50 mm, 500 mm, respectively.

polarizer P1 and quarter-wave plate are tuned for the required incident polarization for isotropic differentiation. With the setup in Supplementary Figure 2(a), the input image can be directly recorded by the camera. In measurement of the output image, the beam experiences an additional reflection and polarization selection process before being recorded by the camera, which is still located at the back focal plane of L2.

In measurement of Figs. 4(d) and 4(g) in the main text, the full circle patterns on the mask have diameters of 1000 μm, 750 μm, 500 μm, 250 μm, 150 μm, 100 μm and 50 μm, respectively. Since the imaging system here performs a demagnification, the full circles in the input image hence have diameters of 100 μm, 75 μm, 50 μm, 25 μm, 15 μm, 10 μm and 5 μm, respectively. Some full circles have very small sizes, which makes the direct observation difficult. Therefore, as shown in Supplementary Figure 2(c), we use an additional imaging system consisting of L3 and L4 to magnify the output image. Specifically, for observation of the smallest full circles with diameters of 10 μm and 5 μm, we use an imaging system with a magnification of 10 and the results are shown as the insets in Figs. 4(d) and 4(g) in the main text.
